# Pivotal Impacts of Retrotransposon Based Invasive RNAs on Evolution

**DOI:** 10.3389/fmicb.2017.01957

**Published:** 2017-10-10

**Authors:** Laleh Habibi, Hamzeh Salmani

**Affiliations:** Department of Medical Genetics, School of Medicine, Tehran University of Medical Sciences, Tehran, Iran

**Keywords:** evolution, retrotransposon, invasive RNA, pseudogene, DNA structure

## Abstract

RNAs have long been described as the mediators of gene expression; they play a vital role in the structure and function of cellular complexes. Although the role of RNAs in the prokaryotes is mainly confined to these basic functions, the effects of these molecules in regulating the gene expression and enzymatic activities have been discovered in eukaryotes. Recently, a high-resolution analysis of the DNA obtained from different organisms has revealed a fundamental impact of the RNAs in shaping the genomes, heterochromatin formation, and gene creation. Deep sequencing of the human genome revealed that about half of our DNA is comprised of repetitive sequences (remnants of transposable element movements) expanded mostly through RNA-mediated processes. ORF2 encoded by L1 retrotransposons is a cellular reverse transcriptase which is mainly responsible for RNA invasion of various transposable elements (L1s, Alus, and SVAs) and cellular mRNAs in to the genomic DNA. In addition to increasing retroelements copy number; genomic expansion in association with centromere, telomere, and heterochromatin formation as well as pseudogene creation are the evolutionary consequences of this RNA-based activity. Threatening DNA integrity by disrupting the genes and forming excessive double strand breaks is another effect of this invasion. Therefore, repressive mechanisms have been evolved to control the activities of these invasive intracellular RNAs. All these mechanisms now have essential roles in the complex cellular functions. Therefore, it can be concluded that without direct action of RNA networks in shaping the genome and in the development of different cellular mechanisms, the evolution of higher eukaryotes would not be possible.

## Introduction

Finding the primary molecule that was responsible for the initiation of life on Earth is the goal of many studies in the field of evolution. Regarding the “central dogma,” the DNA has been a candidate for the name of the molecule of life. However, fans of the “RNA world theory” explain how life could have been started by the RNAs. The discovery of RNAs with enzymatic activity (Ellington and Szostak, 1990; Robertson and Joyce, 1990; Tuerk and Gold, 1990) and the chemical features of different RNAs—along with the widespread viruses using RNA as their only genetic material—are some clues that help scientists describe the RNA world hypothesis (Pressman et al., 2015). In this theory, it is postulated that RNA and RNA-like molecules, which could fold into a three-dimensional structure with catalytic activities, had played central metabolic roles in the ancient world (Bass and Cech, 1984). Additionally, the double feature of tRNAs to bind with the genetic codes in one loop and their specific binding to amino acids in another stem could further confirm the central role of this molecule in early evolution (Lee et al., 2000; Saito et al., 2001; Murakami et al., 2003; Chumachenko et al., 2009). In this review, we have briefly discussed the importance of the intracellular RNAs in the DNA expansion and its role in shaping the genome to create higher order structures and mechanisms throughout the course of evolution.

## Types of RNAs and Intracellular Invasive RNAs

RNAs had been primarily known as the mediators of the gene expression. However, the different types of RNAs with various roles in the eukaryotic and prokaryotic cells have been discovered. Based on their functions, these molecules can be categorized into four different types: (1) Encoding RNAs that contain the codons for the synthesis of polypeptides. (2) Structural RNAs [ribonucoeoproteins (RNPs)] that incorporate into the structure of some proteins; thus, they could have played an essential role in maintaining the steady feature and activity of these proteins (Cech and Steitz, 2014). (3) Catalytic RNAs (ribozymes), associated with proteins (RNPs), and mainly involved in the formation of peptide bonds in the peptidyl transferase center of ribosomes, site specific cleavage, ligation of RNAs, and mRNA splicing (Weinger et al., 2004; Keating et al., 2010; Wilson et al., 2016). (4) Regulatory RNAs (riboregulators), which include the non-coding RNAs with various sequences and sizes. These RNAs could regulate the gene expression by targeting mRNAs, leading to the modification of the rRNA, repressions of transposons, and also involved in X-inactivation, chromatin remodeling, and DNA methylation to repress the transcription (Lippman et al., 2004; Esteller, 2011; Cech and Steitz, 2014).

Apart from these functional molecules, the eukaryotic cells also contain RNAs that are exclusively transcribed to be incorporated into the genome by a mechanism called reverse transcription. This process is mainly involved in the construction of telomere (Autexier and Lue, 2006; Lewis and Wuttke, 2012), formation of pseudogenes (Tutar, 2012; Milligan and Lipovich, 2015), and expansion of retrotransposon (Kassiotis and Stoye, 2016). In all these cases, the intracellular RNAs (which we have called “invasive RNAs” in this paper) could be transformed to cDNA in the nucleus and inserted into the genome through the double strand breaks in the DNA. Generally, three types of invasive RNAs can be considered in the eukaryotic cells. Some of these RNAs have been evolved to form specific genomic constructions, such as the telomerase RNA component (TERC), which functions as a template for the extension of telomeres at the end of the eukaryotic chromosomes (Ozturk et al., 2017). Invasive RNAs transcribed from the retrotransposons do not seem to play any pivotal roles in a cells’ lifecycle, but have been highly effective during evolution (Cordaux and Batzer, 2009). The DNA might also be attacked by functional RNAs. These RNAs are not naturally invasive, but could be transformed into cDNA by intracellular reverse transcriptase (RTs) and result in the formation of pseudogenes (Tutar, 2012).

The RTs are the key enzymes for RNA invasion. Telomerase and ORF2 (reverse transcriptase produced by retrotransposon) are the two known functional RTs in the eukaryotic cells (Meyer et al., 2017). The role of telomerase is confined to the construction of telomeres by using a specific RNA (TERC) as a template (Lewis and Wuttke, 2012); however, ORF2 uses cytoplasmic RNAs and retroelement transcripts to create pseudogenes and cause retrotransposon expansion respectively (Wei et al., 2001). Interestingly, in some eukaryotes, the retroelement-related RT is responsible for the elongation of the telomere (Biessmann et al., 1992).

## Invasive RNAs Originated from Retrotransposons: Structural and Functional Roles

**FIGURE 1:**
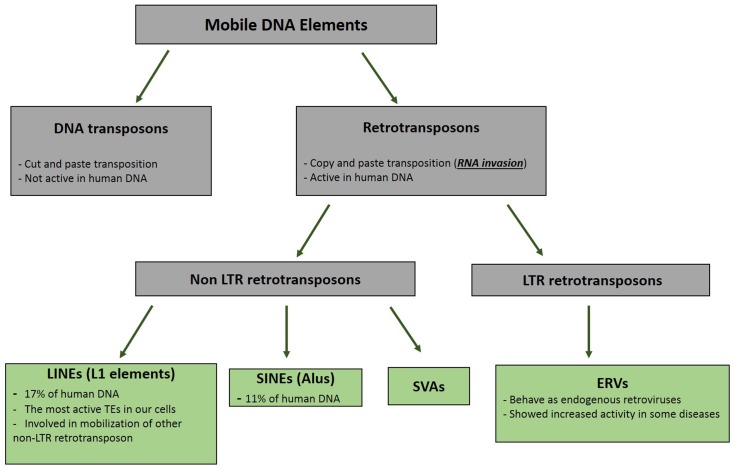
Pedigree of Mobile DNA Elements. Transposable elements are categorized in two distinct groups based on their mode of mobilization. DNA transposons move by cut and paste mechanism, however, retrotransposons (retroelements) mobility are mediated by RNAs. Activity of DNA transposons had been fully shut down during evolution, whereas retroelements still show activities in different types of our cells.

Retrotransposons are groups of mobile DNA elements [transposable elements (TEs)] that copy and paste themselves using the RNA molecules (**Figure 1**). As mentioned in the previous section, these RNA molecules are naturally invasive and are basically transcribed to be randomly inserted into the genome and increase the copy numbers of the retrotransposons (Goodier and Kazazian, 2008). All groups of TEs had been active during the early evolution; however, their selfish and mutagenic movements have resulted in the limitation of their activities to specific types of retrotransposons in the modern human (Marchetto et al., 2013). Long Interspersed Elements (LINE, L1) are the most active retroelement in our cells. It is estimated that the human DNA contains around 500,000 copies of the L1 retrotransposon; however, only 80–100 copies of these elements have maintained their mobility (Goodier and Kazazian, 2008). The structure of a complete L1 element includes a promoter located in the 5′ UTR region, an open reading frame (ORF) 1 gene that encodes the RNA binding protein, ORF2 gene that produces a protein with both endonuclease and reverse transcriptase activity in the two different domains, and a 3′ UTR providing poly-A-tail for the L1 RNA (Goodier and Kazazian, 2008). The RNA polymerase II apparatus is responsible for the production of the L1 RNA (Burns and Boeke, 2012). The transcribed RNA is then transported to the cytoplasm to produce the ORF1 and ORF2 proteins. This invasive RNA in the complex with ORF1 and ORF2 is transported to the nucleus, where it invades the DNA using endonuclease and reverse transcriptase activity of the ORF2 protein by a mechanism called target prime reverse transcription (TPRT) (Cost et al., 2002). The invasive RNAs produced by other retrotransposons (Alu and SVAs) are inserted into the genome through the function of the L1 proteins (Raiz et al., 2012).

**FIGURE 2:**
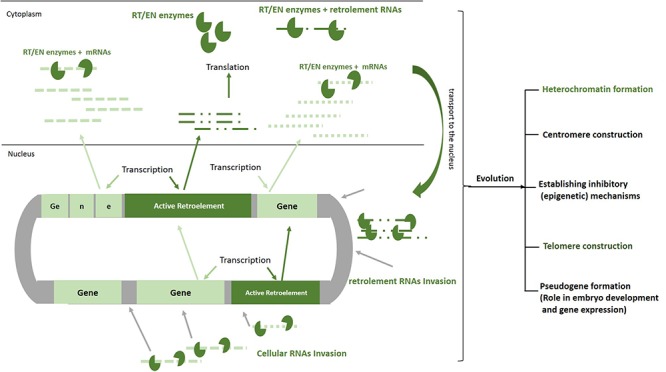
Roles of RNA invasion in shaping human genome. Active retroelements in early evolution have been able to actively transpose and increase their copy number by means of their natural invasive RNAs. Retroelements RNAs similar to mRNAs are transcribed and translated by cellular apparatus. The proteins that are encoded by these elements (EN/RT) can bind to cellular RNAs as well as retroelements RNAs, transport them to the nucleus, create DNA breaks, make cDNA, and finally pasting a copy of each RNA in to the genome. Although the movements of these mobile elements are now inhibited remarkably in our cells, 100 million years of their activities have resulted in formation of heterochromatin, centromere, telomere, and pseudogenes. In order to decrease deleterious effects of retrotransposition, inhibitory mechanisms such as DNA methylation, heterochromatinization, and miRNA production have been established by host cell. EN/RT, endonuclease/reverse transcriptase.

It seems that more than the other TEs, the retrotransposons have a greater impact on changing the structure of the DNA and developing specific cellular mechanisms through 100s million years of evolution (Habibi et al., 2015). The retroelement RNA invasions that occurred most often early during evolution have been caused by the genomic expansion and when the DNA is given the space to create structures, such as heterochromatin and centromere (Nigumann et al., 2002) (**Figure 2**). The human genome project revealed that more than half of our DNA is comprised of non-coding regions. Further evaluation showed that these parts of our genome, which mainly construct the heterochromatin, centromeres, telomeres, and gene spacers, include repetitive sequences comprising the remnants of the retrotransposition events (Lander et al., 2001). The importance of the heterochromatin and centromeres in the gene expression, senescence, embryo development, and cell cycle in the eukaryotes has been found in different studies (Eberl et al., 1993; Harmon and Sedat, 2005; Hammoud et al., 2009; Chandra et al., 2015). Therefore, one can conclude that without the actions of these ancient invasive RNAs, our cells would not perform genomic expansion to form the heterochromatin region, centromeres, telomeres, introns, or regulatory elements, and would remain in the prokaryotic phase. On the other hand, the RT enzyme produced by the retroelements could transform the functional cytoplasmic RNAs into invasive molecules to create pseudogenes (**Figure 2**). This process was essential in the doubling of genes and generation of new genes with different functions throughout the course of evolution (Tutar, 2012). Additionally, it has been shown that the small interfering RNAs transcribed from these pseudogenes might interact with the functional genes in the eukaryotic cells (Ewing, 2017).

Although retrotransposons had been important in the shaping and evolution of the eukaryotic genome, the selfish mobility of these elements would be harmful for DNA integrity and cell viability (Symer et al., 2002). During the retrotransposons’ lifecycle, the invasion of the RNAs by means of the endonuclease/RT enzyme could break the genes, disrupt the open-reading frames, and, finally, affect the production of proteins (Dupuy et al., 2001). The excessive activity of endonuclease produced by the retroelements could also induce excessive DNA double strand breaks (Chen et al., 2006). On the other hand, the promoter region of these elements might also be copied and inserted near the genes and thus influence the quantity and quality of the gene expression (Cordaux and Batzer, 2009). Different lines of studies have shown the increased levels of L1 retrotransposition in different types of cancers (Shpyleva et al., 2017), schizophrenia (Bundo et al., 2014; Doyle et al., 2017), autism (Shpyleva et al., 2017), and Rett syndrome (Muotri et al., 2010), emphasizing the pathogenic role of these elements in the human cells. Regarding these potential threats, the eukaryotic cells have developed repressive mechanisms, including epigenetic modifications (DNA methylation, heterochromatinization), miRNAs, and piRNAs expressions, to inhibit and control the activity of the TEs (mainly retrotransposons) (Habibi et al., 2015). All these repressive pathways have other roles at present rather than the retrotransposons repression inside the cells. Therefore, we can emphasize that the embryo development, differential gene expression, cell differentiation, and specifications would not have occurred without the development of repressive mechanisms against intracellular invasive RNAs.

## Conclusion

Various types of RNAs have been discovered that play a role in the different aspects of the gene expression. Here, we have described another kind of RNAs that are transcribed to invade the DNA and increase their source (retroelement) copy number. These ancient RNAs have a pivotal role in increasing the size of the DNA, establishing heterochromatin, centromeres, telomeres, methylation processes, epigenetic mechanisms, miRNA production, etc. through 100 million years of evolution.

Regardless of the advantageous evolutionary roles; the activities of retrotransposons and their invasive RNAs are highly inhibited in fully differentiated cells (Wissing et al., 2012) since such invasions could be harmful for the genomic integrity of evolved cells. However, different lines of studies showed increased retroelements movements in neural precursor cells (Muotri et al., 2005), embryonic stem cells (Kano et al., 2009) as well as germ cells (Georgiou et al., 2009). One could suggest two ideas for this exceptional high activity of the retrotransposons; (1) in all these cells we are facing to vast changes in epigenetic status of DNA including hypomethylation which could remove the lock of the retroelements and give them chance to increase their movements as side effect of epigenetic changes. These random RNA insertions might result in neurodevelopmental disorders (McConnell et al., 2017) and different kind of cancers (Kano et al., 2009). (2) These increased retrotransposition might do have functional role such as memory storage in neurons (Habibi et al., 2009) and involving in the survival of the organism (Sciamanna et al., 2011). Totally, all these aspects of intracellular invasive RNAs life cycle could show the importance of these elements in creating complex organisms during the evolution.

## Author Contributions

LH: collected data, wrote the paper, designed and drew figures; HS: collected data.

## Conflict of Interest Statement

The authors declare that the research was conducted in the absence of any commercial or financial relationships that could be construed as a potential conflict of interest.
